# Longitudinal association of body mass index with lung function: The CARDIA Study

**DOI:** 10.1186/1465-9921-9-31

**Published:** 2008-04-04

**Authors:** Bharat Thyagarajan, David R Jacobs, George G Apostol, Lewis J Smith, Robert L Jensen, Robert O Crapo, R Graham Barr, Cora E Lewis, O Dale Williams

**Affiliations:** 1 Dept of Laboratory Medicine and Pathology, University of Minnesota, Minneapolis, Minnesota, USA; 2 Division of Epidemiology, School of Public Health, University of Minnesota, Minneapolis, Minnesota, USA; 3 Institute for Nutrition Research, University of Oslo, Oslo, Norway; 4 Abbott Laboratories, Chicago, Illinois (based on work done as a student at Division of, Epidemiology, School of Public Health, University of Minnesota, Minneapolis, Minnesota, USA; 5 Feinberg School of Medicine, Northwestern University, Chicago, Illinois, USA; 6 LDS Hospital, Salt Lake City, Utah, USA; 7 Division of General Medicine, Department of Medicine and Department of Epidemiology, Columbia University Medical Center, New York, New York, USA; 8 Division of Preventive Medicine, Department of Medicine, University of Alabama at, Birmingham, Birmingham, Alabama, USA

## Abstract

**Background:**

Lung function at the end of life depends on its peak and subsequent decline. Because obesity is epidemic in young adulthood, we quantified age-related changes in lung function relative to body mass index (BMI).

**Methods:**

The Coronary Artery Risk Development in Young Adults (CARDIA) study in 1985–86 (year 0) recruited 5,115 black and white men and women, aged 18–30. Spirometry testing was conducted at years 0, 2, 5 and 10. We estimated 10 year change in FVC, FEV_1 _and FEV_1_/FVC according to baseline BMI and change in BMI within birth cohorts with initial average ages 20, 24, and 28 years, controlling for race, sex, smoking, asthma, physical activity, and alcohol consumption.

**Measurements and Main Results:**

Participants with baseline BMI < 21.3 kg/m^2 ^experienced 10 year increases of 71 ml in FVC and 60 ml in FEV_1 _and neither measure declined through age 38. In contrast, participants with baseline BMI ≥ 26.4 kg/m^2 ^experienced 10 year decreases of 185 ml in FVC and 64 ml in FEV_1_. FEV_1_/FVC increased with increasing BMI. Weight gain was also associated with lung function. Those who gained the most weight over 10 years had the largest decrease in FVC, but FVC increased with weight gain in those initially thinnest. In contrast, FEV_1 _decreased with increasing weight gain in all participants, with maximum decline in obese individuals who gained the most weight during the study.

**Conclusion:**

Among healthy young adults, increasing BMI in the initially thin participants was associated with increasing then stable lung function through age 38, but there were substantial lung function losses with higher and increasing fatness. These results suggest that the obesity epidemic threatens the lung health of the general population.

## Background

Many studies find that lung function, as described by the forced expiratory volume in one second (FEV_1_) and/or forced vital capacity (FVC), is inversely correlated with general, pulmonary, and cardiovascular mortality and morbidity [1-3]. FEV_1 _and FVC at the end of life is a function of lung growth during childhood, peak function in early adulthood, and subsequent decline related to aging and insults such as cigarette smoking, air pollution, and occupational exposures [4-8]. Peak lung function in early adulthood is related to gender, race/ethnicity, cigarette smoking, exposure to environmental tobacco smoke and particulate air pollution [7-9]. In addition, lung function is decreased by excess body fatness after adjusting for other factors such as age, height, race, sex, asthma and smoking status in populations that are at risk for reduced lung function [10-19]. However, in the one study that has evaluated the association between BMI and lung function in the general population, the median age was 41 years [20]. No study has evaluated the association between BMI and future lung function in young adulthood.

In addition to increases in body weight with age [21], there are widespread population secular trends of increasing obesity [22]. In the US, the prevalence of obesity, defined as a body mass index (BMI) >30 kg/m^2^, increased from 12% in 1992 to 17.9% in 1998 and to 19.8% in 2000, across all age groups, races, genders and educational levels [23,24]. A recent paper has shown that the prevalence of obesity has increased from 10.9% in 1996 to 22.1% in 2001 in young adults aged 19–26 years [25]. This obesity epidemic may cause a population-wide worsening of lung function.

In the presence of secular and age-related increases in weight and obesity, the goals of the present study were to quantify age-related changes on FVC, FEV_1_, and the FEV_1_/FVC ratio according to baseline BMI and BMI changes in a large, generally healthy, cohort of black men, white men, black women, and white women followed for 10 years. Our hypotheses were (1) greater BMI during young adulthood is inversely related to lung function measures later in life and (2) the effect of change in BMI on future lung function is dependent on the participant's BMI at baseline such that an increase in BMI increases lung function among those who were thin at baseline, but decreases lung function among those with high baseline BMI.

## Methods

### Participants and Measurements

The data used in these analyses were collected in the Coronary Artery Risk Development In Young Adults (CARDIA) study, a multi-center cohort study occurring in the US. The cohorts were recruited from the general population, mostly by telephone, randomly sampled from a prepaid health plan in Oakland, CA and from populations in Birmingham, AL, Chicago, IL, and Minneapolis, MN. The response rate was approximately 50%, which was considered acceptable given the required long term commitment to the study. The detailed methods, instruments and quality control procedures are described in other published reports [26,27]. In 1985–86 (year 0), 5,115 black and white men and women were recruited for the year 0 examination; 4,624 were reexamined in 1987–88 (year 2); 4,352 in 1990–91 (year 5); 4,086 in 1992–93 (year 7); and 3,950 in 1995–96 (year 10). At year 0, CARDIA included approximately equal numbers of participants who were black and white, men and women, aged 18–24 and 25–30, and had more than or less than or equal to high school education [26,27]. We excluded 58 participants who were outside the 18 through 30 age range at year 0, 7 women who were pregnant at baseline, and anyone missing baseline lung function, BMI, physical activity, alcohol intake, or smoking, leaving 4,734 participants for analysis. Of these, 4,277 attended year 2, 4,043 attended year 5, and 3,668 attended year 10. We excluded 147 observations in women who were pregnant at followup measurement of lung function, since pregnancy might influence both BMI and lung function, but included observations in those same women when not pregnant.

Clinic attendance was somewhat higher at the year 10 exam among whites (82%) than among blacks (73%). The participants lost to follow-up after years 0, 2, or 5 did not differ significantly in most of their year 0 characteristics when compared with those observed at year 10. Specifically, both mean FVC and FEV_1 _at year 0 did not differ significantly across those whose last examination attended was year 0 (n = 203), 2 (n = 232), 5 (n = 221), 7 (n = 410), or 10 (n = 3668).

### Measures

Body weight was measured in light clothing to the nearest 0.1 kg with a calibrated balance beam scale, height without shoes was measured to the nearest 0.5 cm using a vertical ruler, and BMI (kg/m^2^) computed.

Demographic characteristics, lifestyle habits, and medical history were collected by self-report using a questionnaire. Physical activity was measured using an interviewer-administered questionnaire [28] concerning the frequency of participation in 13 different activities during the past 12 months. Because participants were not asked specifically about duration of physical activity, exact energy expenditure cannot be estimated and the activity is expressed approximately in "Exercise Units" (EU). A score of 100 EU is roughly equivalent to participation in activities such as a vigorous exercise class or bicycling faster than 10 miles per hour, two or three hours a week for six months of the year. Average weekly alcohol intake was determined separately for beer, wine, and liquor. Smoking status was categorized into four groups: never smokers, ex-smokers, current smokers of ≤ 15 cigs/day, and current smokers of >15 cigs/day. Asthma diagnosis [29] was made at a given examination if the subject was taking asthma medication (usually based on examination of medicine containers) or self-report of a medical diagnosis of asthma (not asked at year 5). The asthma variable had three categories: asthma diagnosed before the beginning of the study, asthma diagnosed during the study and people that never had asthma diagnosed either before or during the study.

Lung function was measured using a Collins Survey 8-liter water sealed spirometer and an Eagle II Microprocessor (Warren E. Collins, Inc., Braintree, MA). Standard procedures of the American Thoracic Society [30] were followed at all examinations. Daily checks for leaks, volume calibration with a 3-liter syringe and weekly calibration in the 4–7 liter range were undertaken to minimize methodological artifacts between exams. We analyzed FVC and FEV_1 _as the maximum of five satisfactory maneuvers and represented as percent of predicted [12,31-34]. In almost all cases, the maximum and second highest maneuvers agreed to within 150 ml.

Year 0 (baseline) BMI, divided into quartiles, was the primary predictor variable. The use of standard NHLBI BMI based adiposity categories to categorize the distribution of BMI in this population resulted in unequal distribution of the population in each category and prevented the detailed evaluation of lung function in thin participants at baseline (Table 1); furthermore, participants changed categories during follow-up. Hence study specific year 0 (baseline) BMI quartile cutpoints were used as the primary predictor variable. However, the percentage of people progressing to different obesity categories (as defined by standard NHLBI cutoffs: normal, < 25 kg/m^2^; overweight, ≥ 25 kg/m^2 ^– < 30 kg/m^2^; and obese, ≥ 30 kg/m^2^) within each baseline BMI category over a 10 year period was calculated [35]. Change in BMI was evaluated as an additional predictor variable. Participants were divided into three age groups: 18–21 years, 22–26 years, and 27–30 years based on their year 0 age. The effect of BMI on lung function was evaluated in these 3 age groups separately.

**Table 1:** Comparison of classification using NHLBI BMI cutpoints with that using the CARDIA baseline BMI quartiles

	**Quartiles of baseline BMI [**n (% in row)]			
**Categories of baseline BMI based on NHLBI BMI cutpoints**	**Q1 <21.3 kg/m**^2^	**Q2 21.3–<23.4 kg/m**^2^	**Q3 23.4–<26.4 kg/m**^2^	**Q4 ≥ 26.4 kg/m**^2^

<18.5 kg/m^2^	193 (100)	0 (0)	0 (0)	0 (0)
18.5–24.9 kg/m^2^	990 (35)	1184 (41)	683 (24)	0 (0)
25–29.9 kg/m^2^	0 (0)	0 (0)	500 (45)	621 (55)
≥ 30 kg/m^2^	0 (0)	0 (0)	0 (0)	563 (100)

### Statistical Methods

We considered that methodological differences might exist between examinations, reflecting small changes in spirometry procedures that occurred by using different technicians across examinations, despite the formal procedures remaining the same. As the first analytic step, we estimated such methodological differences adjusting for race, gender, age, age^2^, height, and height^2 ^by subtracting the mean lung function value for participants of a given age at a later exam from the mean lung function value for other participants of the same age at an earlier examination, then averaging over ages (age-matched calendar time differences) using the method of Jacobs et al. [36]. Relative to year 10 measurements, we added 53 ml, 54 ml, and 16 ml to the predicted FVC at year 0, 2, and 5, respectively; added 6 and 21 ml to the predicted FEV_1 _at years 0 and 2, and subtracted 25 ml from the predicted FEV_1 _at year 5; and subtracted 0.94, 0.55, and 0.91 units from 100* the predicted FEV_1_/FVC ratio at the respective years.

Analyses of lung function and BMI relationships in three narrow age ranges allowed us to separate the cross-sectional and longitudinal relationships as people went through different phases of lung development, plateau, and decline [9,37-39]. Longitudinal changes in lung function over 10 years, as estimated by FVC, FEV_1_, and FEV_1_/FVC at years 0, 2, 5, and 10 were estimated within each age group across different baseline BMI quartiles. Using the lung values corrected for methodological differences, a repeated measures regression model (SAS PROC MIXED) adjusted for current age, time, race, sex, height, age group category, smoking status, physical activity, and alcohol intake (all at baseline) and baseline prevalence and incidence of asthma [9,36,38,39] was used to estimate the association of baseline BMI with lung function. The covariates were selected a priori based on their associations with the variables of interest. Linearity assumptions and goodness of fit were verified by examining the sequence of mean dependent variable values at each age within each BMI category in reference to the fitted lines. Goodness of fit was adequate (data not shown). Serial correlation was modeled as compound symmetry. We estimated the effect of concurrent change in BMI from year 0 to year 10 on lung function using a repeated measures regression with change in the lung parameter as the dependent variable (3 repeats: year 2 – year 0, year 5 – year 0, and year 10 – year 0), and baseline BMI quartiles, change in BMI in 5 categories, and their interaction as the independent variables of interest. The 4 categories of least change in BMI stratified a large number of people, while the highest category allowed a closer evaluation of change in lung function among those who gained a considerable amount of weight (≥ 6 kg/m^2^). In analyses evaluating the association between change in BMI and change in lung function, we added as covariates change in smoking status, change in physical activity, and change in alcohol intake. People who were heavier at baseline tended to gain more weight over 10 years than did people who were lighter. Therefore this additional model evaluated how much of the lung function and BMI relationship persisted after accounting for subsequent weight change. It also evaluated the relationship of lung function to change in BMI itself. For comparison with the model of change since baseline, we also examined a transition model [40] in which the repeated changes were for year 2 – year 0, year 5 – year 2, and year 10 – year 5. Here a BMI increase ≥ 6 kg/m^2 ^was rare given that the maximum time interval between examinations was 5 years, so the highest BMI change category was ≥ 2.5 kg/m^2^.

## Results

### Description of Study Population

**Figure 1:**
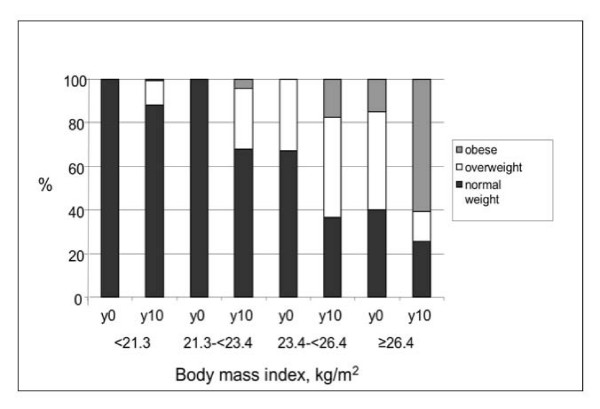
**Presence of overweight and obesity according to NHLBI cutpoints** (overweight body mass index (BMI) 25–29.9 kg/m^2^, obese BMI ≥ 30 kg/m^2^), by quartile of baseline BMI; and progression over 10 years.

The study sample at year 0 was aged 24.9 ± 3.6 years (Table 2). There were 1017 in the 18–21 year old birth cohort (mean age 19.6 years), 1842 in the 22–26 year old cohort (mean age 24.1), and 1875 in the 27–30 year old cohort (mean age 28.5). By design, the participants were evenly distributed among race-sex groups. Thirty nine percent had no education past high school. 2225 had never smoked and never had asthma either prior to year 0 or during the 10 years of study. Quartile cut points for year 0 BMI, computed before exclusion for missing covariates, were: 25th percentile 21.2 kg/m^2^, median 23.4 kg/m^2^, and 75th percentile 26.4 kg/m^2^. A higher percentage of participants were black in the highest BMI quartile as compared to lower BMI quartiles (64% vs. 46%). Progression in NHLBI BMI-based adiposity categories [35] is depicted in Figure 1. Overall, the mean increase in BMI over 10 years was 3.0 ± 3.5 (SD) kg/m^2^. Category cut points 0, 1, 2.5, and 6 kg/m^2 ^for 5 categories of change in BMI were selected to represent weight loss and gradations of weight gain. The increase in BMI tended to be larger, the higher the initial BMI (Table 3).

**Table 2:** Characteristics of the participants at year 0 according to baseline BMI quartiles, the CARDIA study, 1985–96

	**Quartiles of Baseline BMI**			
	**Q1 <21.3 kg/m**^2^	**Q2 21.3–<23.4 kg/m**^2^	**Q3 23.4–<26.4 kg/m**^2^	**Q4 ≥ 26.4 kg/m**^2^

	**(n = 1117)**	**(n = 1191)**	**(n = 1215)**	**(n = 1211)**
**Age (Years)**	24.3 (3.7)	24.7 (3.5)	25.1 (3.6)	25.3 (3.5)
**FVC (L)**	4.02 (0.88)	4.40 (0.96)	4.58 (1.06)	4.19 (1.05)
**FEV_1 _(L)**	3.38 (0.68)	3.64 (0.75)	3.74 (0.82)	3.44 (0.82)
**FEV_1_/FVC**	85 (7)	83 (6)	82 (6)	83 (6)
**Physical activity (exercise units)**	395 (275)	462 (299)	459 (317)	375 (290)
**Alcohol (mg/day)**	10.6 (19.0)	12.9 (22.8)	13.5 (21.0)	11.4 (22.4)
**Education (% ≤ high school)**	39.8	37.6	35.6	42.6
**Race (Blacks) (%)**	44.1	45.1	48.8	64.4
**Sex (Male) (%)**	32.6	51.2	57.5	43.0
**Prevalence of asthma at year 0 (%)**	9.0	9.2	10.9	10.2
**Cumulative incidence of asthma during 10 years of follow-up (%) **	6.8	4.9	5.4	8.1
**Prevalence of ex-smokers (%)**	14.2	13.0	15.1	12.1
**Prevalence of current smoking ≤ 15 cigs/day (%)**	21.7	21.6	19.1	20.9
**Prevalence of current smoking > 15 cigs/day (%)**	9.9	9.9	10.0	9.8

* Variables were measured at year 0 unless otherwise indicated. Values are means (standard deviations in parentheses) or percentages.

**Table 3:** Distribution of categories of 10 year change in BMI across baseline BMI quartiles

	**Quartiles of baseline BMI [**n (% in column)]			
**Categories of Change in BMI**	**Q1 <21.3 kg/m**^2^	**Q2 21.3–<23.4 kg/m**^2^	**Q3 23.4–<26.4 kg/m**^2^	**Q4 ≥ 26.4 kg/m**^2^

≤ 0 kg/m^2^	115 (14)	149 (17)	146 (16)	138 (15)
0.1–0.9 kg/m^2^	137 (17)	128 (15)	103 (12)	63 (7)
1–2.4 kg/m^2^	232 (29)	227 (26)	187 (21)	136 (15)
2.5–5.9 kg/m^2^	254 (31)	270 (31)	323 (36)	292 (33)
≥ 6 kg/m^2^	70 (9)	102 (12)	132 (15)	263 (29)

### Lung Function and BMI: Association between year 0 BMI and lung function

**Figure 2:**
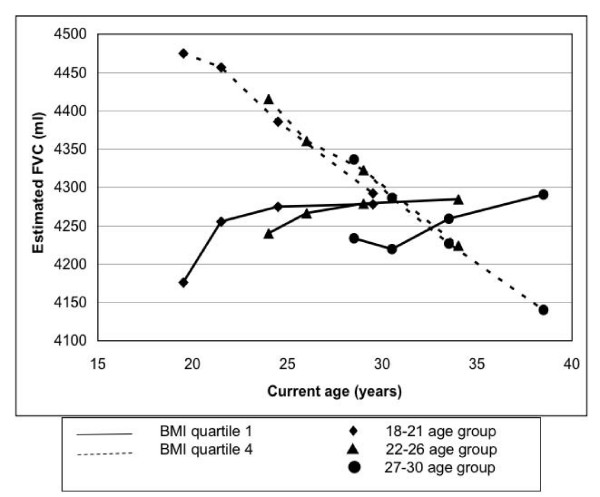
**FVC in year 0 BMI quartiles across three birth cohorts: 18–21 years, 22–26 years, and 27–30 years at baseline, based on repeated measures linear regression analysis and adjusted for race, sex, current age, smoking status at year 0, asthma status, time, physical activity score at year 0, and alcohol consumption at year 0. **The slope of FVC across time becomes increasingly negative with increasing year 0 BMI (p trend < 0.0001).

The change in FVC over a 10 year period differed across baseline BMI quartiles (p < 0.0001). Average 10 year FVC change was 71, 19, -72, and -185 ml in the lowest through highest BMI quartile (Table 4), with the increase in the low BMI participants being more pronounced in the youngest birth cohort (Figure 2). The estimated mean FVC generally increased for 5 years, then plateaued in all birth cohorts in the thinnest people at baseline (Figure 2). A pattern of increase and plateau is seen from mean age 19.6 years, with no decrease in FVC through mean age 38.5 years (Figure 2, oldest birth cohort, year 10). In contrast, for those in the highest BMI quartile FVC decreased continuously over the same time period in all birth cohorts. People in the second quartile of baseline BMI displayed a tendency to increase FVC over 10 years, but less so than in quartile 1 (data not shown) and people in the third quartile of baseline BMI displayed a tendency to decrease FVC, but less so than in quartile 4 (data not shown).

**Table 4:** Estimated* 10 year change in FVC (mL), FEV_1 _(mL) and FEV_1_/FVC (%) across baseline BMI quartiles, overall and among never smokers who did not have asthma at baseline or during the study

**Quartiles of baseline BMI**	**10-year Change in lung function value **		
**All participants**	**FVC**	**FEV1**	**FEV1/FVC**
<21.2 kg/m^2^	71 (-43 – 184)	60 (-38 – 159)	-0.07% (-1.40% – 1.26%)
21.3–<23.4 kg/m^2^	19 (-94 – 133)	18 (-79 – 116)	0.29% (-1.03% – 1.62%)
23.4–<26.4 kg/m^2^	-72 (-186 – 41)	-28 (-125 – 70)	1.00% (-0.33% – 2.33%)
≥ 26.4 kg/m^2^	-185 (-298 – -71)	-64 (-161 – 34)	2.03% (0.71% – 3.36%)
p trend	<0.0001	<0.0001	<0.0001
			
**Never smoker, never asthma**			
<21.2 kg/m^2^	129 (-37 – 294)	76 (-62 – 215)	-0.84% (-2.63% – 0.95%)
21.3–<23.4 kg/m^2^	78 (-87 – 244)	29 (-109 – 168)	-0.60% (-2.39% – 1.18%)
23.4–<26.4 kg/m^2^	-18 (-184 – 147)	-28 (-166 – 111)	0.05% (-1.73% – 1.84%)
≥ 26.4 kg/m^2^	-138 (-304 – 27)	-47 (-186 – 91)	1.60% (-0.19% – 3.39%)
p trend	<0.0001	<0.0001	<0.0001

* Estimated FVC, FEV_1 _and FEV_1_/FVC were obtained using a repeated measures linear regression model to estimate lung function values over a 10 year period across baseline BMI quartiles after adjusting for current age, (current age)^2^, race, gender, study center, height, (height)^2^, baseline age group, smoking status, asthma status, physical activity and alcohol intake all measured at baseline (year 0).

**Figure 3:**
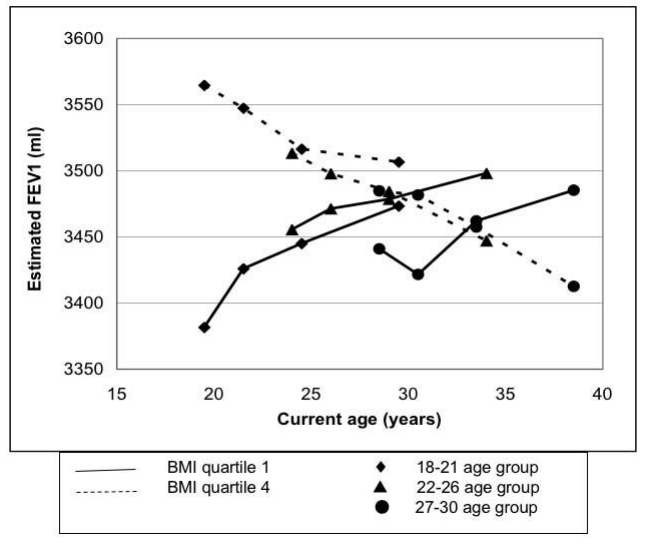
**FEV_1 _in year 0 BMI quartiles across three birth cohorts: 18–21 years, 22–26 years, and 27–30 years at baseline, based on repeated measures linear regression analysis and adjusted for race, sex, current age, smoking status at year 0, asthma status, time, physical activity score at year 0, and alcohol consumption at year 0.** The slope of FEV_1 _across time becomes increasingly negative with increasing year 0 BMI (p trend < 0.0001).

The change in FEV_1 _over a 10 year period also differed across baseline BMI quartiles (p < 0.0001). The FEV_1 _change was 60, 18, -28, and -64 ml in the lowest through highest BMI quartiles (Table 4), respectively, with the increase in the low BMI participants being more pronounced in the youngest birth cohort and no suggestion of a decline in FEV_1 _through age 38 in the lowest BMI quartile (Figure 3).

**Figure 4:**
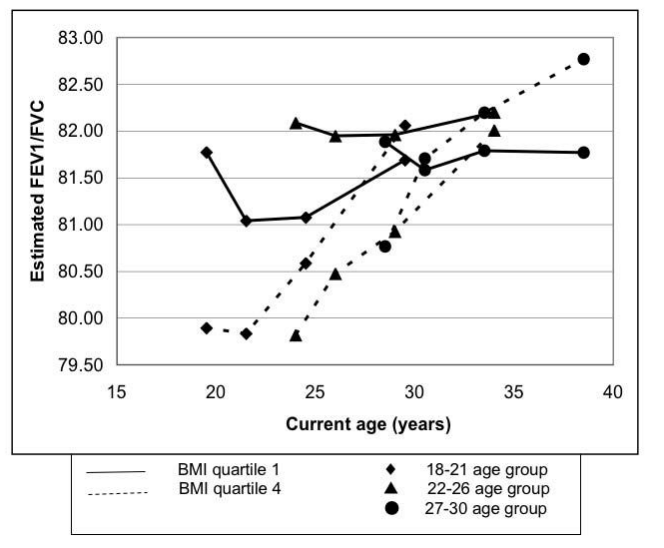
**FEV_1_/FVC in year 0 BMI quartiles across three birth cohorts: 18–21 years, 22–26 years, and 27–30 years at baseline, based on repeated measures linear regression analysis and adjusted for race, sex, current age, smoking status at year 0, asthma status, time, physical activity score at year 0, and alcohol consumption at year 0.** The slope of FEV_1_/FVC across time becomes increasingly positive with increasing year 0 BMI (p trend < 0.0001).

In contrast to the FVC and FEV_1_, estimated mean FEV_1_/FVC tended to decrease in the thinnest participants for the first 5 years and then increase over the next 5 year period as compared to a continuous increase in the participants in the highest baseline BMI quartile (Table 4, Figure 4); p for changes in the ratio across BMI categories was < 0.0001 within each age group and did not vary significantly by age group. Age-adjusted mean change in FEV_1_/FVC (averaged across the 3 birth cohorts) was -0.07, 0.29, 1.00 and 2.03 in the lowest to highest BMI quartiles, respectively (Table 4), with the initial decrease in the low BMI participants being more pronounced in the youngest cohort.

Findings were similar among the 2225 participants at year 0 who were never smokers and did not have asthma at any time during the study (Table 4). Race and gender did not significantly modify the association between year 0 BMI and any of the lung function variables (data not shown).

### Lung Function and BMI: Association Between Change in BMI and Change in Lung Function

Change in BMI was a significant predictor of FVC, FEV_1_, and FEV_1_/FVC over the 10 year study period. The direction of change in lung function according to change in BMI was dependent on year 0 BMI, but not on birth cohort (p < 0.0001 for the interaction of change in BMI and baseline BMI, Table 5). Averaging across the 3 birth cohorts, FVC increased over the study period in the lowest BMI quartile across all categories of change in BMI, although the increase was least in those who lost weight (5 ml) or who gained > 6 kg/m^2 ^(5 ml) as compared to a increase in FVC in those who gained 1–5.9 kg/m^2 ^(15–65 ml) (p for difference for those who lost weight as compared to those who gained 2.5–5.9 kg/m^2 ^< 0.0001) (Table 5). In contrast, in individuals in the highest baseline BMI quartile FVC increased in those who lost weight (22 ml) or those who gained 0.1–0.9 kg/m^2 ^(15 ml), but decreased progressively as weight gain increased, reaching a loss of 264 ml in those who gained > 6 kg/m^2 ^(p for difference <0.0001). Within each category of change in BMI, baseline BMI remained a significant predictor of FVC (p value < 0.0001).

**Table 5:** Estimated* 10 year change in FVC (mL), FEV_1 _(mL) and FEV_1_/FVC (%) across different categories of change in BMI within baseline BMI quartiles

	Quartiles of baseline BMI *^+^			
	**<21.3 kg/m**^2^	**21.3–<23.4 kg/m**^2^	**23.4–<26.4 kg/m**^2^	**≥ 26.4 kg/m**^2^
**2, 5 and 10 year FVC change (p interaction < 0.0001)**				
**Categories of Change in BMI**	**Q1**	**Q2**	**Q3**	**Q4**
≤ 0 kg/m^2^	5 (-14 – 24)	32 (14 – 49)	49 (32 – 67)	22 (4 – 40)
0.1–0.9 kg/m^2^	15 (1 – 30)	22 (8 – 37)	22 (8 – 37)	15 (0 – 31)
1–2.4 kg/m^2^	53 (38 – 67)	19 (4 – 33)	-13 (-27 – 2)	-63 (-80 – -46)
2.5–5.9 kg/m^2^	65 (46 – 83)	1 (-17 – 18)	-69 (-85 – -53)	-146 (-161 – -130)
≥ 6 kg/m^2^	5 (-37 – 46)	-96 (-132 – -60)	-178 (-208 – -148)	-264 (-286 – -243)
**2, 5, and 10 year FEV_1 _change (p interaction < 0.0001)**				
**Categories of Change in BMI**	**Q1**	**Q2**	**Q3**	**Q4**
≤ 0 kg/m^2^	-38 (-56 – -19)	-18 (-35 – -2)	-5 (-21 – 11)	-11 (-22 – 6)
0.1–0.9 kg/m^2^	-31 (-45 – -17)	-24 (-38 – -10)	-22 (-36 – -8)	-30 (-45 – -16)
1–2.4 kg/m^2^	-30 (-44 – -16)	-60 (-73 – -46)	-66 (-80 – -52)	-81 (-97 – -64)
2.5–5.9 kg/m^2^	-54 (-71 – -36)	-95 (-111 – -78)	-121 (-137 – -106)	-136 (-151 – -121)
≥ 6 kg/m^2^	-110 (-149 – -70)	-147 (-181 – -112)	-201 (-230 – -173)	-216 (-236 – -195)
**2, 5, and 10 year FEV_1_/FVC change (p interaction < 0.0001)**				
**Categories of Change in BMI**	**Q1**	**Q2**	**Q3**	**Q4**
≤ 0 kg/m^2^	-0.96 (-1.30 – -0.63)	-1.03 (-1.33 – -0.74)	-0.99 (-1.29 – -0.70)	-0.70 (-1.01 – -0.40)
0.1–0.9 kg/m^2^	-1.03 (-1.28 – -0.78)	-1.03 (-1.28 – -0.78)	-1.01 (-1.26 – -0.75)	-1.01 (-1.27 – -0.74)
1–2.4 kg/m^2^	-1.85 (-2.10 – -1.61)	-1.71 (-1.95 – -1.46)	-1.24 (-1.49 – -0.98)	-0.68 (-0.97 – -0.39)
2.5–5.9 kg/m^2^	-2.59 (-2.91 – -2.28)	-2.18 (-2.47 – -1.89)	-1.55 (-1.82 – -1.27)	-0.51 (-0.78 – -0.25)
≥ 6 kg/m^2^	-3.30 (-4.01 – -2.59)	-2.10 (-2.72 – -1.48)	-1.60 (-2.12 – -1.08)	-0.11 (-0.48 – 0.26)

* Estimated FVC, FEV_1 _and FEV_1_/FVC were obtained from a repeated measures linear regression model that evaluated the change in lung function from baseline over 2, 5 and 10 years across different baseline BMI quartiles after adjusting for current age, (current age)^2^, race, gender, study center, height, (height)^2^, age group, smoking status, asthma status, and alcohol intake all measured at baseline (year 0), change in smoking status, change in physical activity and change in alcohol intake (all over 2, 5 and 10 years).

Averaging across all 3 birth cohorts, FEV_1 _decreased in all baseline BMI quartiles across all categories of change in BMI. In the lowest baseline BMI quartile, the decrease was lower in those who lost weight (-38 ml) or gained minimal weight (-31 ml) as compared to those who gained > 6 kg/m^2 ^(-110 ml) during the same period (Table 5) (p for difference for >6 kg/m^2 ^as compared to those who lost weight = 0.001). Individuals in the highest baseline BMI quartile also lost increasing amounts of FEV_1 _with increasing change in BMI (Table 5) (p for difference for > 6 kg/m^2 ^as compared to those who lost weight < 0.0001). The magnitude of loss of FEV_1 _was higher in the highest baseline BMI quartile as compared to the lowest baseline BMI quartile.

The FEV_1_/FVC decreased among all participants. The decrease in FEV_1_/FVC among the lowest baseline BMI quartile was higher with increasing weight gain (p for difference for > 6 kg/m^2 ^as compared to those who lost weight < 0.0001) as compared to the decrease in FEV_1_/FVC among the highest baseline BMI quartile with increasing weight gain (p for difference = 0.01 for those who gained > 6 kg/m^2 ^as compared to those who lost weight) (Table 5).

The transition model using year 2 – year 0, year 5 – year 2, and year 10 – year 5 as repeats but otherwise parallel to the model shown in Table 5 showed a similar pattern for each lung function measure to that shown in Table 5, but lung function changes for each BMI change category were generally smaller than in Table 5, consistent with the shorter exposure intervals being modeled (2, 3, and 5 year intervals in the transition model, compared to 2, 5, and 10 year intervals in Table 5).

Restricting to participants who were never smokers and never had asthma during the study, the direction and magnitude of association between change in BMI and FVC, FEV_1_, and FEV_1_/FVC was similar to that seen in the entire population (data not shown). This similarity included that change in BMI was a significant predictor of FVC, FEV_1 _and FEV_1_/FVC within each baseline BMI category (p <0.0001 for the interaction of change in BMI and baseline BMI).

## Discussion

We found strong associations between lung function and BMI. As hypothesized, FVC and FEV_1 _generally decreased over a 10 year period both with higher baseline BMI and with increasing BMI over 10 years of follow-up. However, the thinnest people (lowest baseline BMI quartile) gained FVC and lost the least amount of FEV_1 _even as they gained weight during the study. Furthermore, our estimates suggested no clear decline in either FVC or FEV_1 _in the thinnest people even through age 38 regardless of concurrent change in BMI. Plateauing of FVC over 10 years of follow-up was observed in all three baseline age groups, suggesting that the observed evolution of FVC was not an artifact of grouping people who achieve their peak lung function at different times [41].

The finding of a decrease in lung function with increasing baseline BMI is in agreement with several cross-sectional studies that found associations of FVC and FEV_1 _with BMI [10-12,18] and other longitudinal studies that found that weight gain is associated with more rapid loss of lung function [13-17,20]. While many of these studies looked at populations at risk for reduced lung function (smokers [13] steel workers, [13,14] or shipyard workers [18]), our study involved a large, generally healthy, young adult sample whose characteristics were much closer to those of the general population than was the case in the other studies. Contrary to what has been reported, we found maintenance of high levels of lung function in the thinnest people (lowest baseline BMI quartile) even through age 38 [42].

FVC as determined by spirometry reflects total compliance, which has contributions from both the lung and chest wall. The FEV_1 _reflects these same factors plus airway resistance. In a normal healthy population, the decrease in elasticity with age has a greater effect on FEV_1 _as compared to FVC, resulting in a decrease in FEV_1_/FVC. However, among the participants with high BMI the FEV_1_/FVC is larger and the loss of elasticity has a greater effect on FVC as compared to FEV_1 _resulting in an increase in FEV_1_/FVC in this subgroup. This is substantiated by our results which show an increase in FEV_1_/FVC over 10 years among participants in the highest BMI category. Increasing year 0 BMI and subsequent weight gain within each BMI quartile can decrease FVC and FEV_1 _by decreasing chest wall compliance and/or increasing the circulating levels of cytokines. Increased adiposity has been associated with increased levels of cytokines such as IL-6 and TNF-alpha [43], and decreased levels of adiponectin [43,44], thereby increasing the levels of systemic inflammation, which might in turn negatively affect lung function. We have previously reported from these data worse lung function in those with higher values for plasma fibrinogen [45]. Increases in both FVC and FEV_1 _over 10 years in the lowest year 0 BMI quartile and maintenance of relatively high FVC and FEV_1 _values even in those thin people who reached their mid 30s during the study is contrary to what has been previously described [42]. In addition, FVC increased in the lowest baseline BMI quartile with increasing change in BMI while FEV_1 _decreased minimally. We consider the possibility that the associations between lung function and BMI observed here are not solely due to the mechanical properties of the chest wall. Lower levels of cytokines and less baseline systemic inflammation in people with low baseline BMI may also explain the observed longitudinal increase in FEV_1 _and FVC even when there was a subsequent increase in BMI. For example, a thin person at baseline whose BMI increases by 5 kg/m^2 ^would still only have a BMI of 24 kg/m^2 ^at year 10. However, serial measurements of cytokines and measures of chest wall compliance are not available to adequately address cytokine behavior or chest wall dynamics in the different baseline BMI and BMI change categories.

The increases in FVC and FEV_1 _observed in the lowest baseline BMI quartile were more pronounced in the youngest birth cohort as compared to the other birth cohorts. Since the people in the youngest age group may still be increasing their lung function, increasing BMI in them could preferentially reflect lean mass, which may have a positive effect on lung function early in adult life, as compared to the detrimental effect in older adults where the increase in BMI more likely represents increasing adiposity [46,47]. This is consistent with the results from another study that showed a positive effect of childhood BMI on adult FVC and FEV_1 _[48].

The present study has several strengths, including the large number of participants, their relatively narrow age range at entry, inclusion of blacks and whites and men and women, and the long duration of follow-up including the period in which peak lung function is achieved. It also assured a high quality of data collection through strict quality control across examinations. Because the sample studied by CARDIA included young, healthy people, few individuals were lost due to disease, avoiding survivorship bias [49]. 3146 participants completed all 4 spirometry tests, 1159 completed 3, 502 completed 2, and 285 completed only 1 test. Parallel analyses in the constant cohort (not missing lung function at any of the 4 examinations (n = 3062 after excluding missing covariates) led to similar results (data not shown), indicating that there was not a substantial bias due to missing observations in this study.

Limitations of the current study include biases common to longitudinal study, such as bias introduced due to loss of follow up. This bias is minimized in CARDIA due to the excellent retention of the original cohort and because there was no difference in baseline lung function measures between those who were lost to follow up and those who continued to participate in the study. Standardizing serial lung function measurements requires technician training and careful adherence to written test protocols. We used standardized measurement techniques and trained technicians to perform spirometry measurements over the 10 year period. In spite of these efforts, we observed a secular trend with FVC values obtained at year 10 being lower than those obtained in the earlier time points and FEV_1 _values being higher at year 0 and 2 and lower at year 5 as compared to year 10 values. We adjusted for this trend during our analysis to minimize the effect of this secular trend on the study results.

In conclusion, participants in this study who were thin at age 18–30 did not experience a decline in FVC and FEV_1 _through their mid 30s. In contrast, increasing BMI in heavier people, particularly those who had a BMI ≥ 26.4 kg/m^2^, 79% of whom had become obese by year 10, was associated with a rapid decrease in FVC and FEV_1 _and an almost constant FEV_1_/FVC ratio. Loss of lung function by age 38 was not inevitable in these healthy young adults, although those with highest BMI suffered substantial losses starting as early as age 20. Whatever the predominant mechanism(s) responsible for these changes might be, these data indicate that maximal lung function may be maintained well into the fourth decade of life; and that, in addition to its other effects on health and disease, the obesity epidemic may threaten the lung function and as a consequence the lung health of the general population.

## Competing interests

All the authors of this paper declare that they have no financial or other potential conflicts of interest concerning the subject of this manuscript.

## Authors' contributions

BT performed all analyses and wrote the initial draft of the paper. DJ obtained funding for the project, conceived the question, and directed writing and analysis. GA, LS, RJ, RC, RB, CL, and OW participated in funding, data collection, data analysis and interpretation, and editing. The manuscript was reviewed and approved by the CARDIA Steering Committee. All authors have read and approved the final manuscript.
